# Understanding the effect of social media marketing activity for promoting intention to participate in martial arts

**DOI:** 10.3389/fpsyg.2022.999153

**Published:** 2022-10-24

**Authors:** Ming-Chuan Xie, Yao-Chuan Chang, Chuan-Ming Cai

**Affiliations:** ^1^School of Physical Education and Health, Zhaoqing University, Guangdong, China; ^2^School of Physical Education, Jimei University, Jimei, China

**Keywords:** social media, user experience, attitudes toward martial arts, martial arts attachment, theory of planned behavior

## Abstract

The development of folk martial arts in China has encountered many obstacles and difficulties in promoting the sport. Although there are many martial arts-related groups on WeChat, the largest social media in China, the interaction is not enthusiastic enough and the participation is too low. The main purpose of this study is to understand the impact of social media marketing activities and user experience on the intention of people to participate in martial arts through a quantitative research method. After the literature study, a research model was developed based on Theory of Planned Behavior (TPB), in which the constructs include social media marketing activities, user experience, attitudes toward martial arts, subjective norms, martial arts attachment, and participation intention. The results of the study illustrated that social media marketing activities and user experience had a positive and significant effect on martial arts attitudes, subjective norms, and martial arts attachment *via* Structural Equation Modeling (SEM). Martial arts attitudes, subjective norms, and martial arts attachment had a positive and significant effect on the intention to participate. Finally, based on the results of this study, we propose suggestions for social media marketing activities, user experience, martial arts attachment, attitudes toward martial arts, subjective norms, and martial arts participation intentions for martial arts social media operators, martial arts promotion organizations, and subsequent studies.

## Introduction

Martial art is a form of physical exercise for fitness, strength, and defense that was created by Chinese ancestors and evolved over a long period of time as the Chinese people lived and battled (Trausch, 2018). The demand for martial arts-related competitions, performances, and the video industry has grown significantly. Martial arts is an educational and recreational part of many families’ lives in China as well as in the west and has become an integral part of cultural physical activity (Sun, 2021). Previous research has shown that the promotion of martial arts in the community seems to be inadequate (Huang and Hong, 2018). For example, Zeng and Yang (2021) examined the factors influencing university students’ participation in martial arts. The study mentioned that martial arts education faces many problems, including the varying quality of instructors, the lack of a strict promotion system, and the low number of channels for learning martial arts, which make the development and promotion of martial arts difficult.

Fortunately, the Chinese community still actively promotes martial arts through the social media WeChat martial arts groups (Peng, 2018). However, there is also a low level of community participation, resulting in ineffective promotion of martial arts (Liu et al., 2020). Mak et al. (2022) used social media to assist martial arts learning through video sharing, teaching material exchange, and teacher-student interaction. In addition, in order to accelerate the entry of specific Chinese martial arts events into the Olympic Games, Han et al. (2021) advocate using social media to promote martial arts events and organize martial arts competitions, so that the international martial arts community can understand Chinese martial arts more clearly and more people from different countries can participate in Chinese martial arts sports. Filo et al. (2015) also made the same claim. Although research has confirmed that social media is an effective channel for marketing and promoting the sport (Hazari, 2018). It is the degree of participation of community members that is the key factor in the success of marketing and promotion (Chung et al., 2016; Bazrkar et al., 2021). In other words, increasing community involvement is an important issue in promoting martial arts. Research confirms that social media marketing activities (Choedon and Lee, 2020), user experience (Khan, 2022), sport attachment (Dalangin et al., 2020), and the attitudes and subjective norms of the theory of planned behavior (Leung and Jiang, 2018; Majeed et al., 2021) can influence intention to participate in, purchase, etc.

Also, in Table 1 Measurement items, the subjective norms scale refers to the scale of same variable by Jordan et al. (2018), Bae and Chang (2021), and VG et al. (2021).

In summary, this study aims to increase the participation of martial arts enthusiasts in the martial arts community. In this study, the social media martial arts group members are used as the target population. The effects of social media marketing activities, user experience, martial arts attachment, attitudes toward martial arts, and subjective norms on the intention to participate in the martial arts community will be investigated (Lau and Chen, 2012). Social media marketing activities are the various martial arts information, performances, and competitions that members share or interact with in the social media platform. User experience refers to the extent to which martial arts group members perceive the social media to be enjoyable and easy to use. Martial arts attachment refers to the strength of the emotional connection of martial arts group members to the various martial arts activities of the community. Furthermore, Theory of Planned Behavior (TPB) is a model of behavioral decision making proposed by Ajzen (1985), which consists of the components of behavioral attitudes, subjective norms, perceived behavioral control, and behavioral intention. Through the statistical analysis results, the study hopes to propose practical practices to enhance the participating intention of martial arts community members and provide a reference for social media group operators, martial arts promotion units, and subsequent related studies.

## Literature review and research hypotheses

### Social media marketing activities

Jong et al. (2021) analyzed how social media use affects work performance. The study mentions that social media can be used to meet people with similar interests, to connect with friends you know, and to meet new friends. In order to better represent the content of social media marketing, scholars have proposed social media marketing activities (Chen and Lin, 2019; Wibowo et al., 2020). Kim and Ko (2012) defined social media marketing activities as activities that aim to promote the understanding of goods on social media. Seo and Park (2018) analyzed the impact of social media marketing activities on user response and brand equity in the aviation industry. The study defines social media marketing activities as a type of advertising on an online platform. In summary, this study defines social media marketing activities as a variety of martial arts marketing activities that involve interacting, collaborating, or sharing content through social media. Ruangkanjanases et al. (2020a) investigated the factors influencing the intention to continue using social media. The study showed that usage satisfaction and perceived usefulness had a positive effect on intention to continue using. Chen et al. (2012) explored several factors that influence the intention to continue using Web 2.0, and found that all the social factors influence the intention to continue using Web 2.0.

### Theory of planned behavior

TPB evolved from the theory of reasoned action, which describes and predicts human behavior (Ajzen, 1991). It advocates that attitudes, subjective norms, and perceptual behavioral control act as independent variables that jointly influence behavioral intentions and further influence behavior. Attitude stands for an individual’s negative or positive evaluation of a specific person, event, object, or behavior (Azjen, 1980), and the psychological tendency to express approval or disapproval of a specific behavior (Chen and Hung, 2016). In this study, attitudes toward martial arts were defined as positive or negative evaluations of the marketing and promotional content of the community by martial arts members. The subjective norms are the social pressure experienced by an individual (Ajzen, 1991). Individuals are usually willing to act under the expectations of those who they consider important (Ruangkanjanases et al., 2020b; Chen et al., 2021). In this study, subjective norms were defined as the extent to which members of the martial arts community support their participation in social media by those who they consider important. Behavioral intention is the likelihood of engaging in a specific behavior (Fishbein et al., 1980), and the personal negative or positive feelings about performing the specific behavior (Davis, 1989). In this study, the intention to participate in martial arts was defined as the likelihood that members of the martial arts community would engage in the marketing and promotion of the content.

Sun and Wang (2019) investigated the relationship between social media marketing on product attitudes, subjective norms, and purchase intention. The results indicate that social media marketing positively and significantly affects attitudes and subjective norms towards products. Similar results were found in Cheunkamon et al. (2020). Based on the above studies, this study proposes hypotheses H1 and H2.

*H1*: Social media marketing activities will have a positive and significant effect on attitudes toward martial arts.

*H2*: Social media marketing activities will have a positive and significant effect on subjective norms.

### Martial arts attachment

In the study of product purchase intention, product attachment is defined as the intensity of the connection between consumers and brands (Park et al., 2014). Based on the above study, this study defines martial arts attachment as the strength of emotional attachment to martial arts of social media members. VanMeter et al. (2018) examined how marketers can increase consumer attachment to brands through social media marketing. The results indicate that social media marketing affects consumers’ attachment to the brand. Based on the above study, this study proposes hypothesis H3 as follows.

*H3*: Social media marketing activities will have a positive and significant effect on martial arts attachment.

### User experience

User experience is the personal perception and responses in the process of using a service (Jang and Han, 2022), the extent to which users have an emotional and cognitive impact (Röttger et al., 2017; Wibowo et al., 2020). This study defines user experience as the extent to which users perceive the martial arts community to be enjoyable and easy to use. Chang and Lin (2015) used customers of creative companies in Taiwan as the target population and showed that customer experience positively influences attitudes and subjective norms. Anshu et al. (2022) used online shopping consumers as the experimental subjects, and the results indicated that shopping attitude would be positively and significantly affected. In summary, this study proposes hypotheses H4 and H5 that user experience will influence attitudes toward martial arts and subjective norms.

*H4*: User experience will have a positive and significant effect on attitudes toward martial arts.

*H5*: User experience will have a positive and significant effect on subjective norms.

Tsai (2016) investigated the relationship between travel experience, place attachment, and behavioral intention to consume local food among Taiwanese travelers. The results showed that the local travel experience positively influenced their local attachment. Vada et al. (2019) analyzed whether travel experience and hedonic well-being could influence travelers’ place attachment. The results showed that travel experience affects their place attachment. Based on the above study, hypothesis H6 is proposed in this study.

*H6*: User experience will have a positive and significant effect on martial arts attachment.

Kim and Kim (2021) investigated the effect of attitude and subjective norms on members’ intention to continue participating in a fencing club in South Korea. The results indicated that attitudes and subjective norms had a positive and significant effect on the intention to continue participation. Similar results have been found in some other studies (Huang et al., 2022). Based on the above studies, this study proposes hypotheses H7 and H8.

*H7*: Attitudes toward Martial arts will have a positive and significant effect on participating intention.

*H8*: Subjective norms will have a positive and significant effect on participating intention.

Jin et al. (2020) investigated the decision-making process of travelers’ participation in Guilin, China, and they also investigated the effect of place attachment and impression of tourist places on revisit intention. Prior research found that place attachment had a positive and significant effect on revisit intention (Jin et al., 2020). Petravičiūtė et al. (2021) used Lithuanian online visitors as the target population, and this study also investigated the effect of luxury brand attachment on purchase intention and found that luxury brand attachment affects purchase intention. Based on the above study, this study proposes hypothesis H9 as follows.

*H9*: Martial attachment will have a positive and significant effect on participating intention.

### Research framework

**Figure 1:**
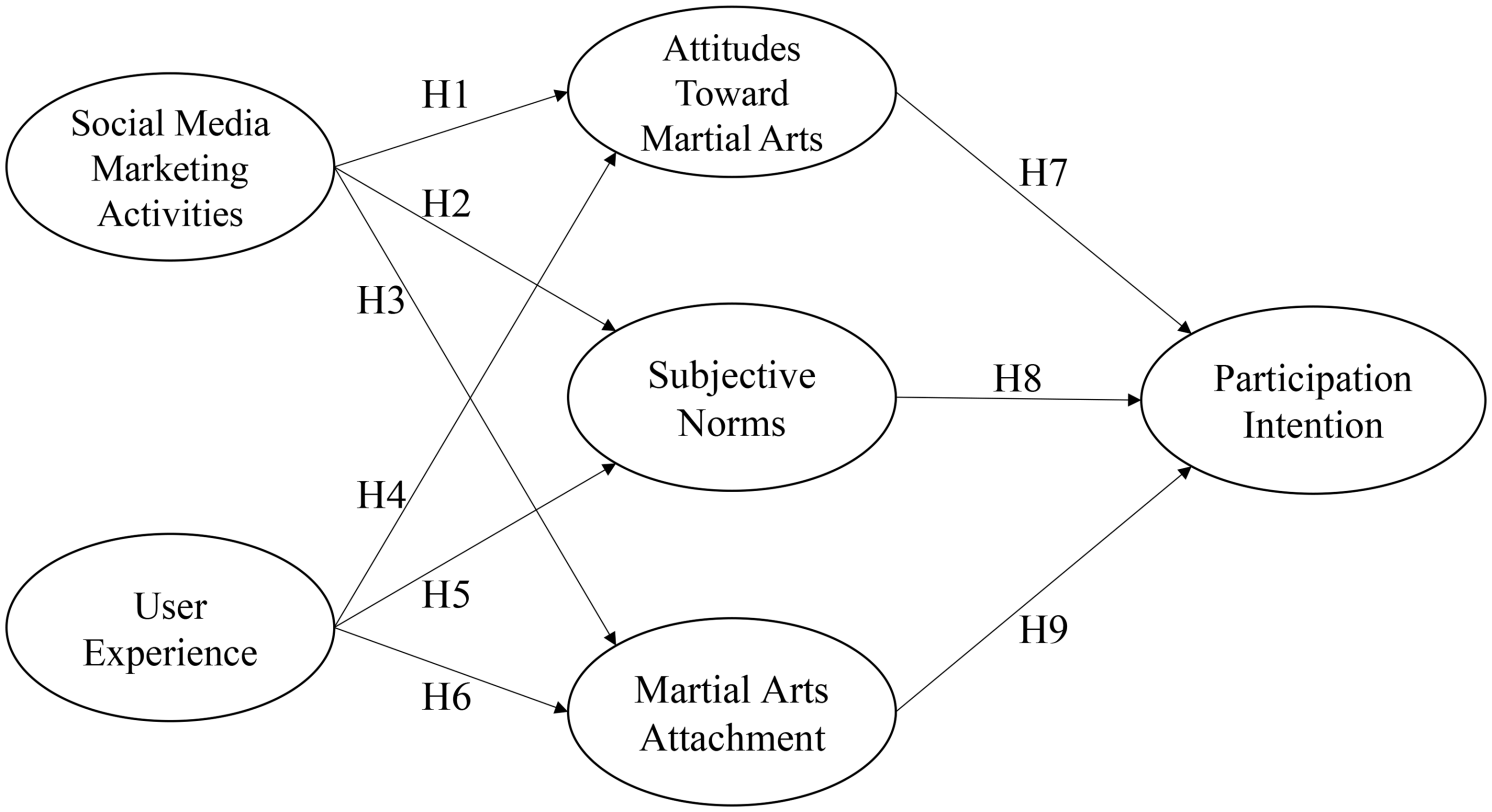
Research framework.

The main purpose of this study is to determine the intention to participate in martial arts. The social media marketing activities and user experience are the independent variables, and martial arts attachment, attitudes toward martial arts, and subjective norms are the mediating variables to investigate their effects on the intention to participate in martial arts. The structure of this study was developed from the literature review as shown in Figure 1.

## Methods

### Research subjects and data collection

This study investigated the factors influencing the intention to participate in martial arts, and the target population was the members of the martial arts group in WeChat, China. An online electronic questionnaire was distributed for data-gathering. A total of 462 questionnaires were collected during the period of 1 May 2022, to 5 May 2022. After removing the invalid questionnaires, the total of valid ones was 451. Based on the proposed formula for sample size requirements in Petravičiūtė et al. (2021), the total number of people interested in martial arts was 384 with a statistical confidence level of 95% and a confidence interval of 5%.

### Measurement scales

This study’s structural framework includes six constructs and the scales. The scales consisted of two sections. Section one collected demographic data, including gender, age, and education level. Section two employed a nominal five-point Likert scale (ranging from 1 “strongly disagree” to 5 “strongly agree”) to collect participants’ opinions on the six variables. Participants answered the survey questions according to their self-perception. Professional scholars were invited to review and comment on the questionnaire items after the scale design. The scales and measurement items are shown as Table 1.

**Table 1:** Measurement items.

Construct	Measures	Items	Source
Social media marketing activities	SMMA1	I use martial arts social media because it is fun.	Ebrahim (2020), Wibowo et al. (2020)
	SMMA2	I use martial arts social media because the content is interesting.	
	SMMA3	I use martial arts social media because I can share martial arts information with others.	
	SMMA4	I use martial arts social media because I can have conversations and share comments.	
	SMMA5	I use martial arts social media because it is easy to post my opinions.	
	SMMA6	I use martial arts social media because it shows the most up-to-date content.	
	SMMA7	I use martial arts social media because it shows the latest martial arts information.	
	SMMA8	I use martial arts social media because the group provides a customized search for martial arts information.	
	SMMA9	I use martial arts social media because it provides a customized service.	
	SMMA10	I would like to pass on information about martial arts competitions, performances, and learning from the martial arts community to my friends.	
	SMMA11	I am willing to repost martial arts community content on my IG, Facebook, and Line groups.	
User experience	UE1	The postings on martial arts social media tried to catch my attention.	Hsu and Tsou (2011), Chen and Lin (2015)
	UE2	It is very interesting to participate in martial arts social media, such as liking and sharing comments.	
	UE3	Martial arts social media posting attempts get me into a certain mood.	
	UE4	Martial arts social media gets me excited.	
	UE5	Martial arts social media tries to make me think about the way I live my life.	
	UE6	Martial arts social media reminds me of the activities I can do.	
	UE7	Martial arts social media tries to pique my interest.	
	UE8	Martial arts social media tries to pique my curiosity.	
	UE9	Martial arts social media tries to make me think about my relationship with fellow martial artists.	
	UE10	I can connect with other martial artists through martial arts social media.	
Martial arts attachment	MA1	I really enjoy using martial arts social media.	Hwang et al. (2019), Tasci et al. (2022)
	MA2	Using martial arts social media has brought me a lot of joy and happiness.	
	MA3	I am very attached to martial arts social media.	
	MA4	Using martial arts social media is my favorite thing to do on vacation.	
	MA5	There is no other social media that can provide the same experience as martial arts social media.	
Attitudes toward martial arts	AT1	I can establish the correct concept of martial arts after using martial arts social media.	Alić et al. (2014), Yang et al. (2020)
	AT2	I can learn new martial arts skills after using martial arts social media.	
	AT3	I can learn new martial arts knowledge after using martial arts social media.	
	AT4	I can feel relaxed after using martial arts social media.	
	AT5	I can experience the fun of martial arts after using martial arts social media.	
Subjective norms	SN1	People who are important to me think I can use martial arts social media.	Jordan et al. (2018), Bae and Chang (2021)
	SN2	People who are important to me support my use of martial arts social media.	
	SN3	People who are important to me understand my use of martial arts social media.	
	SN4	People who are important to me agree with my use of martial arts social media.	
	SN5	People who are important to me also use martial arts social media.	
Intention to participate in martial arts	PI1	I plan to participate in the activities of martial arts social media in the future.	Lin (2006), Sharma and Klein (2020)
	PI2	I intend to participate in the activities of martial arts social media in the future.	
	PI3	I expect to participate in the activities of martial arts social media in the future.	

### Research method

This study used the questionnaire survey method. The validated samples were analyzed by SPSS 23.0, and descriptive statistics and inferential statistics were examined. The data analysis methods included (1) Frequency distribution: the frequency distribution and percentage of gender, age, and educational level of the sample; (2) Construct validity analysis: The standardized factor loading of confirmatory factor analysis was used to examine the reliability of the questions and constructs. The average variance extracted was used to examine the convergent validity of all constructs. The square roots of average variance extracted were used to compare the correlation coefficients between the constructs to check the discriminant validity between the constructs; (3) Structural equation model testing: including the model fit testing and the hypotheses testing between the constructs through path analysis.

## Data analysis

### Descriptive analysis

The target population of this study was members of the Chinese WeChat martial arts community. There were 451 valid samples. In terms of gender, most of the members were male, with a total of 267 participants accounting for 59.2%. In terms of age, most of the members were 31–40 years old, with a total of 268 participants accounting for 59.4%. In terms of education level, the majority were university students, with a total of 222 participants accounting for 49.2%, as shown in Table 2.

**Table 2:** Frequency distribution.

Category	Group	Number of counts	Percentage
Gender	Male	267	59.2
	Female	184	40.8
Age	Below 20	4	0.9
	21–30	41	9.1
	31–40	268	59.4
	Above 41	138	30.6
Educational level	Below high school	158	35.0
	University	222	49.2
	Master or above	71	15.7

### Construct reliability and validity

Cronbach’s alpha is a widely used measure of the reliability of a survey instrument, which is acceptable at a range of 0.7 or above (Nunnally, 1994). As shown in Table 3, the standardized factor loadings of each scale in this study were greater than 0.6, and the reliability of all constructs was greater than 0.7. And, the values of Cronbach’s alpha and composite reliability of all constructs are greater than 0.7. It can be concluded that all constructs in this study have met reliability. Moreover, all average variance extracted were greater than the value of 0.5 suggested by Hair et al. (1998).

**Table 3:** Confirmatory factor analysis.

Construct	Std.	SMC	Cronbach’s alpha	CR	AVE
Social media marketing activity	0.712–0.853	0.507–0.728	0.906	0.948	0.624
Sense	0.746–0.829	0.557–0.687	0.810	0.832	0.624
Feel	0.766–0.811	0.587–0.658	0.807	0.826	0.612
Think	0.672–0.858	0.452–0.736	0.798	0.817	0.601
Act	0.631–0.803	0.398–0.645	0.764	0.786	0.553
Relate	0.898–0.943	0.806–0.889	0.935	0.941	0.842
User experience	0.656–0.819	0.430–0.671	0.856	0.871	0.575
Attitude toward martial arts	0.670–0.944	0.449–0.891	0.905	0.922	0.708
Subjective norms	0.697–0.906	0.486–0.821	0.889	0.903	0.653
Martial arts attachment	0.645–0.863	0.416–0.745	0.856	0.874	0.583
Participating intention	0.607–0.941	0.368–0.885	0.865	0.884	0.611

Std. = standardized factor loading; SMC, squared multiple correlation; CR, composite reliability; AVE, average variance extracted.

This study adopted bootstrapping method to evaluate the 95% confidence intervals of the correlations of different constructs as a basis for judgement (Torkzadeh et al., 2003), and none of the 95% confidence intervals include 1, indicating the acceptance of discriminant validity between the constructs (as shown in Table 4). Therefore, the results demonstrate that the proposed model has the sufficient discriminant validity.

**Table 4:** Discriminant validity.

				Bias-corrected		Percentile	
Parameter			Estimate	Lower	Upper	Lower	Upper
SMMA	<−->	UE	0.603	0.370	0.778	0.335	0.762
SMMA	<−->	AT	0.527	0.380	0.642	0.392	0.648
SMMA	<−->	SN	0.622	0.431	0.79	0.438	0.799
SMMA	<−->	MA	0.694	0.499	0.869	0.51	0.879
SMMA	<−->	RI	0.670	0.507	0.878	0.502	0.864
UE	<−->	AT	0.705	0.542	0.937	0.552	0.948
UE	<−->	SN	0.554	0.347	0.727	0.339	0.721
UE	<−->	MA	0.662	0.434	0.822	0.387	0.808
UE	<−->	RI	0.472	0.300	0.654	0.283	0.635
AT	<−->	SN	0.357	0.202	0.497	0.213	0.508
AT	<−->	MA	0.388	0.262	0.509	0.262	0.509
AT	<−->	RI	0.416	0.288	0.550	0.283	0.548
SN	<−->	MA	0.761	0.584	0.916	0.587	0.917
SN	<−->	RI	0.507	0.313	0.687	0.326	0.702
MA	<−->	RI	0.537	0.365	0.744	0.365	0.746

SMMA, social media marketing activity; UE, user experience; AT, attitude toward martial arts; SN, subjective norms; MA, martial arts attachment; PI, participating intention.

#### Structural model inspection

In this study, all nine fitness indicators are within the tolerable range, indicating that the agreement between the predicted and actual observed values of the model in this study is acceptable (Table 5).

**Table 5:** Model fit criteria and the test results.

Fit indices	Allowable range	Measurement model	Structural model	Model adaptation
Chi-square		1884.55	1896.825	
Degree of freedom		183	185	
Comparative Fit Index (CFI)	>0.9	0.971	0.969	Passed
Root Mean Square Error of Approximation (RMSEA)	<0.08	0.045	0.046	Passed
Tucker-Lewis Index (TLI)	>0.9	0.969	932	Passed
Goodness-of-Fit Index (GFI)	>0.9	0.942	0.939	Passed
Normed-Fit Index (NFI)	>0.9	0.942	0.939	Passed
χ^2^/df	<3	1.932	1.947	Passed
Adjusted Goodness-of-Fit Index (AGFI)	>0.8	0.935	0.932	Passed

As shown in Table 6, Hypothesis 1, the standardized path coefficient of social media marketing activity on attitude toward martial arts is 0.210 (*p* = < 0.001), so H1 is supported. Hypothesis 2, the standardized regression coefficient of social media marketing activity on subjective norms is 0.421 (*p* < 0.001), so H2 is accepted. Hypothesis 3, the path coefficient of social media marketing activity on martial arts attachment is 0.452 (*p* < 0.001), so H3 is verified. Hypothesis 4, the standardized regression coefficient of user experience on attitude toward martial arts is 0.491 (*p* < 0.001), so H4 is supported. Hypothesis 5, the path coefficient of user experience on subjective norms is 0.336 (*p* < 0.001), so H5 is accepted. Hypothesis 6, the standardized regression coefficient of user experience on martial arts attachment is 0.416 (*p* < 0.001), so H6 is accepted. Hypothesis 7, the standardized path coefficient of attitude toward martial arts on participating intention is 0.191 (*p* < 0.001), so H7 is verified. Hypothesis 8, the standardized path coefficient of subjective norms on participating intention is 0.179 (*p* < 0.001), so H8 is supported. Hypothesis 9, the standardized path coefficient of martial arts attachment to participating intention is 0.374 (*p* < 0.001), so H9 is accepted.

**Table 6:** Regression coefficient.

Endogenous variable	Exogenous variable	Regression weight	Standard error	*z*-value	Srandardized path coefficient	*p*-Value	*R* ^2^
AT	SMMA	0.180	0.049	3.676	0.210	0.000	0.415
	UE	0.825	0.120	6.901	0.491	0.000	
SN	SMMA	0.370	0.052	7.091	0.421	0.000	0.470
	UE	0.578	0.108	5.344	0.336	0.000	
MA	SMMA	0.392	0.048	8.159	0.452	0.000	0.615
	UE	0.708	0.105	6.755	0.416	0.000	
PI	AT	0.260	0.068	3.820	0.191	0.000	0.380
	SN	0.238	0.070	3.379	0.179	0.000	
	MA	0.502	0.078	6.421	0.374	0.000	

## Results and discussion

### Academic findings and contributions

After empirical analysis, the results of this study verified that social media marketing activities have positive and significant effects on attitudes toward martial arts, subjective norms, and martial arts attachment. These three direct effects were found to be consistent with previous studies VanMeter et al. (2018), Sun and Wang (2019), and Bi et al. (2021), respectively. In other words, the content of various martial arts promotion and marketing activities shared through the interaction of the martial arts social media groups will positively affect the evaluation of martial arts by the group members. The extent to which members consider important people support their participation in social media activities, and the strength of their emotional connection to the marketing and promotional content of the community.

Second, the results also validated that user experience had a positive and significant effect on subjective norms, martial arts attachment, and attitudes toward martial arts. The first two direct effects were found to be consistent with the results of previous studies Chang and Lin (2015) and Vada et al. (2019), respectively. That is the users’ enjoyment and ease of use of the martial arts community. Such a high level of emotional awareness positively influences the extent to which members consider important people to support their participation in social media activities. Members’ emotional connection to the marketing and promotional content of the community is strengthened.

Furthermore, the results validated that attitudes toward martial arts, subjective norms, and martial arts attachment all had positive and significant effects on the intention to participate in martial arts. These three direct effects were found to be consistent with the results of previous research on the TPB, Kim and Kim (2021), Huang et al. (2022), and attachment-related research, Jin et al. (2020), respectively. In other words, martial arts group members’ evaluation of martial arts, the extent to which members consider important people to support their participation in social media activities, and the strength of their emotional connection to the marketing and promotional content of the community positively influenced their intention to participate in martial arts.

### Managerial and practical implications

We propose practical suggestions based on direct hypotheses of social media marketing activities to improve their effectiveness as follows. First, social content should incorporate interesting or entertaining topics (Wibowo et al., 2020). For example, the social media operators should consider to combine the hottest martial arts movie episodes and martial arts stars into videos or commentaries, and ask members what they think and whether they want to see a sequel. Second, the social media operators should try to ask open-ended questions that stimulate dialogue. For example, what kind of martial arts sport interests you most. Third, organize activities with simple topics with social media users. For example, the social media operators could invite members to vote for martial arts stars so that members can easily participate. Fourth, the social media operators should induce members to invite their friends to group events and give them extra incentives. For example, by referring friends or inviting them to join a martial arts group through an invitation code. When participating in community or physical martial arts competitions or performances, you can get a discount on tickets or receive a participation ticket.

Based on the direct hypothesis findings of user experience and research which points out user experience of social media can be enhanced through videos, pictures, and emotional sharing (Koetz, 2018), this study proposes the following practical suggestions to enhance the user experience of the martial arts community. First, the live streaming of martial arts competitions, teaching and performances gives group members the opportunity to comment and interact directly with the host in real time. In addition, this will also increase the curiosity of the members about the content of the live broadcast, thus increasing their experience and participation. Second, enhance the photo experience of martial arts community members. For example, create highly visually appealing martial arts or interesting martial arts storyline GIFs. Third, create content that touches the emotions of martial arts community members. For example, the growth stories of famous martial arts stars and athletes or their difficult training process.

### Research limitations and future works

This study has some limitations. First, the study was conducted with a sample of Chinese WeChat martial arts group members, and no comparative or differential analysis of other social media was conducted. For example, Weibo, Little Red Book, and Douyin. In other words, the future study must include members of different social media martial arts groups. In addition, the status of martial arts community members may affect their willingness to fill out the questionnaire, e.g., group administrators and members who are more frequently involved in group activities are more willing to fill out the questionnaire. This may affect the validity of the results. Moreover, other factors that affect the intention to participate in martial arts can be included in the future. This will further improve the model to understand the effect of other factors and social media on participation intention. Lastly, this study was conducted only on Chinese martial arts community users. In the future, we can explore the participation of people in different countries and explore the intention to participate in martial arts from a national and regional perspective.

## Data availability statement

The raw data supporting the conclusions of this article will be made available by the authors, without undue reservation.

## Author contributions

M-CX, Y-CC, and C-MC: conceptualization. M-CX and Y-CC: data curation. M-CX and Y-CC: formal analysis. Y-CC and C-MC: investigation. All authors have read and agreed to the published version of the manuscript.

## Conflict of interest

The authors declare that the research was conducted in the absence of any commercial or financial relationships that could be construed as a potential conflict of interest.

## Publisher’s note

All claims expressed in this article are solely those of the authors and do not necessarily represent those of their affiliated organizations, or those of the publisher, the editors and the reviewers. Any product that may be evaluated in this article, or claim that may be made by its manufacturer, is not guaranteed or endorsed by the publisher.
